# Integrative network toxicology and machine learning reveal the potential role of 5-hydroxymethylfurfural in exacerbating pulmonary fibrosis

**DOI:** 10.1371/journal.pone.0361700

**Published:** 2026-11-02

**Authors:** Qinghuan Yang, Fuli Li, Jiani Li, Ting Qin

**Affiliations:** 1 Department of Respiratory Disease, Chongqing Jiulongpo Science City People’s Hospital, Chongqing, China; 2 Department of Neurology, The Second Affiliated Hospital of Chongqing Medical University, Chongqing, China; Kurume University School of Medicine: Kurume Daigaku Igakubu Daigakuin Igaku Kenkyuka, JAPAN

## Abstract

**Background:**

5-Hydroxymethylfurfural (5-HMF), a ubiquitous furan derivative formed during the thermal processing of carbohydrate-rich foods, represents a widespread dietary exposure. However, its potential contribution to idiopathic pulmonary fibrosis (IPF) remains unclear. This study investigated the molecular links between 5-HMF and IPF using an integrated computational and experimental approach.

**Methods:**

IPF-related genes were identified through transcriptomic analysis combining differential expression analysis and weighted gene co-expression network analysis. Candidate targets were obtained by intersecting IPF-associated genes with predicted 5-HMF targets. Functional enrichment analysis, ensemble machine learning, and SHAP interpretation were performed to prioritize candidate targets, while molecular docking was used to explore potential 5-HMF–target interactions. A bleomycin-induced mouse model of pulmonary fibrosis was further used to evaluate the effects of 5-HMF on fibrotic lung injury and the transcriptional expression of prioritized candidate genes.

**Results:**

A total of 1,776 IPF-associated genes and 143 predicted 5-HMF targets were identified, yielding 12 overlapping candidate targets. Enrichment analysis implicated these genes in fibrosis-related pathways, including chemical carcinogenesis–receptor activation and estrogen signaling. Machine learning analysis prioritized seven core targets (CA2, CA4, ESR2, F10, HSP90AA1, PLA2G1B, and PTPRB), all of which accommodated 5-HMF in their orthosteric pockets in blind docking, albeit with weak, non-selective Vina scores (−4.5 to −5.4 kcal/mol). In vivo, 5-HMF significantly exacerbated bleomycin-induced pulmonary fibrosis, as demonstrated by aggravated lung injury, increased collagen deposition, and elevated expression of Hsp90aa1, Pla2g1b, Ca4, F10, Acta2, and Col1a1.

**Conclusion:**

Our findings suggest that 5-HMF may exacerbate fibrotic lung injury through multiple fibrosis-related molecular targets and pathways. These findings provide preliminary evidence for a potential association between 5-HMF exposure and the aggravation of pulmonary fibrosis, while further studies are required to determine whether 5-HMF can independently initiate fibrotic pathology.

## Introduction

Idiopathic pulmonary fibrosis (IPF) is a progressive, irreversible interstitial lung disease with a dismal prognosis, imposing a heavy and growing global health burden [1–3]. With a median survival of merely 3–5 years post-diagnosis, IPF contributes substantially to interstitial lung disease-related mortality worldwide [4]. Its pathogenesis involves a complex interplay of genetic susceptibility, aging, and environmental exposures, leading to repetitive alveolar epithelial cell injury, aberrant fibroblast activation, and excessive extracellular matrix deposition [5]. Among environmental risk factors, chronic exposure to inhaled pollutants and dietary toxicants has emerged as a critical contributor to disease initiation and progression, though the underlying mechanisms remain largely elusive [6,7].

5-Hydroxymethylfurfural (5-HMF) is a furan derivative formed during the thermal processing and storage of carbohydrate-rich foods and beverages, such as coffee, bread, and fruit juices, making it a widespread dietary contaminant [8–10]. It is also generated endogenously through the Maillard reaction and is present in tobacco smoke, contributing to overall human exposure [11]. While 5-HMF has been evaluated for potential genotoxicity and carcinogenicity with conflicting results, emerging evidence suggests it can induce cellular stress responses relevant to fibrotic pathways. Studies indicate that 5-HMF exposure can promote oxidative stress, trigger inflammatory cytokine release, and regulate endoplasmic reticulum stress [12,13]. These processes are central to the pathogenesis of IPF, where epithelial cell dysfunction and a pro-fibrotic microenvironment drive persistent fibrosis [14]. However, systematic investigations into the potential role of 5-HMF in IPF remain scarce.

The advent of network toxicology and machine learning offers a transformative paradigm for deciphering complex chemical-disease interactions [15]. By integrating heterogeneous and high-dimensional molecular data, computational target prediction, and network analysis, these approaches can identify complex relationships across multiple biological layers and prioritize biologically relevant signals that may be difficult to capture using conventional single-target strategies [16]. Such integrative computational approaches have been increasingly applied to identify potential chemical–gene–disease associations and generate testable hypotheses in toxicological research [16,17]. Recent advances have further demonstrated the utility of interpretable machine learning in respiratory toxicology. For example, Li et al. developed deep-learning models for multiple chemical-induced respiratory toxicity endpoints, including pulmonary fibrosis, and incorporated SHAP analysis to improve model interpretability, highlighting the potential of explainable artificial intelligence for chemical respiratory toxicity assessment [18]. However, their predictive performance is inherently influenced by data quality, model generalizability, and interpretability, and computationally prioritized associations should not be regarded as standalone evidence of toxicological causality without biological validation [17]. Therefore, integrating network toxicology and machine learning with subsequent computational and experimental evaluation may provide a hypothesis-generating framework for identifying potential molecular links between environmental exposures and complex diseases.

In the present study, we adopted an integrative computational and experimental strategy to investigate potential molecular links between 5-HMF and pulmonary fibrosis. First, we constructed a multi-source target profile for 5-HMF and integrated it with IPF-related transcriptomic data to identify overlapping candidate targets. Functional enrichment analysis was then used to delineate the involved biological processes and pathways. Subsequently, multiple machine learning algorithms were leveraged to prioritize candidate genes with discriminatory value for IPF, and SHAP analysis was applied to interpret model predictions. Molecular docking was then conducted to explore potential interactions between 5-HMF and the prioritized target proteins. Finally, a bleomycin-induced mouse model of pulmonary fibrosis was used to experimentally evaluate whether 5-HMF exacerbates fibrotic lung injury and to examine the expression of selected candidate targets. Through this integrated framework, we aimed to generate and experimentally evaluate hypotheses regarding potential molecular associations between 5-HMF exposure and the aggravation of pulmonary fibrosis, providing a basis for further investigation of its biological effects and underlying mechanisms.

## Materials and methods

### Acquisition of IPF-related transcriptomic data

Transcriptomic datasets related to IPF were retrieved from the NCBI GEO database. Three datasets (GSE53845, GSE110147, and GSE10667) were integrated to form a discovery (training) cohort for comprehensive analysis. Additionally, two independent datasets (GSE24206 and GSE70866) were selected as external validation cohorts. To alleviate batch effects, a multi-step normalization protocol was employed. First, latent confounding factors within the discovery cohort were estimated and adjusted using Surrogate Variable Analysis (SVA). Subsequently, residual technical batch variations were harmonized across datasets using the ComBat algorithm, which applies a parametric empirical Bayes framework. Principal component analysis (PCA) was utilized to evaluate the efficacy of multi-stage correction, ensuring reduced batch clustering and improved phenotype-associated sample distribution in reduced dimensions. The overall analytical workflow is summarized in Fig 1.

**Fig 1:**
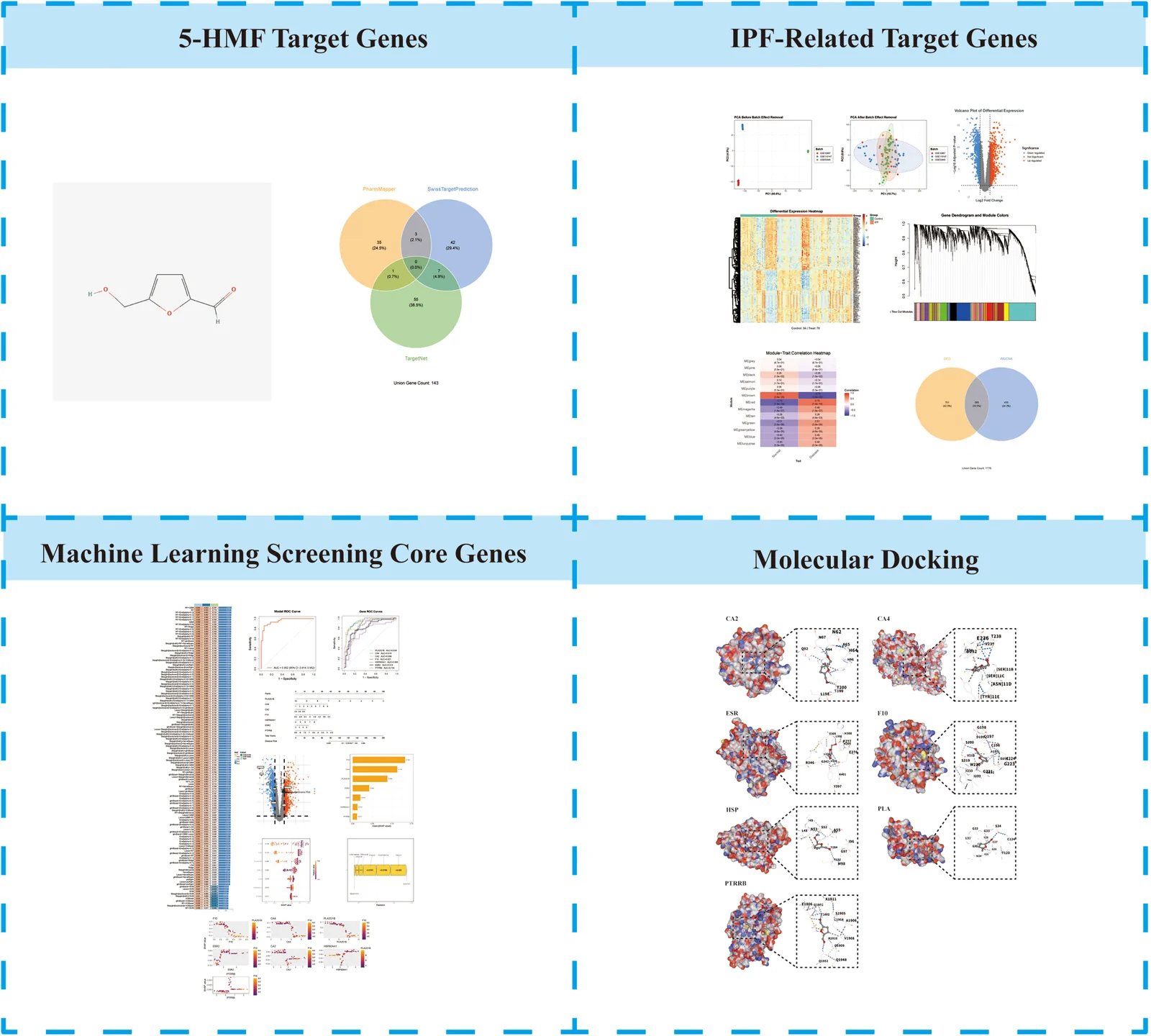
Flowchart of the integrative study design. The schematic diagram illustrates the multi-step analytical framework, encompassing the prediction of 5-HMF targets, identification of IPF-related genes via transcriptomic analysis (DEGs and WGCNA), prioritization of candidate genes for IPF classification using ensemble machine learning, followed by molecular docking analysis of potential 5-HMF–target interactions.

### Acquisition of chemical components and targets of 5-HMF

The chemical characterization and target profiling of 5-HMF were conducted through the integration of multiple authoritative databases. Its physicochemical properties and relevant biological parameters were systematically retrieved from PubMed. The canonical 2D structural representation (SMILES: C1=C(OC(=C1)C=O)CO) was obtained from the PubChem database. Target prediction adopted a three-pronged approach involving three platforms: TargetNet (http://targetnet.scbdd.com) for quantitative structure-activity relationship (QSAR)-based polypharmacology prediction; SwissTargetPrediction (http://www.swisstargetprediction.ch) for chemical genomics-based prediction; and PharmMapper (http://lilab-ecust.cn/pharmmapper) for 3D pharmacophore matching. All predicted targets were limited to the Homo sapiens proteome.

### Weighted gene co-expression network analysis (WGCNA) and DEG identification

Differential expression analysis was performed on the integrated IPF training set using the limma package, with statistical significance thresholds set at P<0.05 and $|{\text{log}}_{\text{2}}FC|>\text{0}.\text{585}$. Simultaneously, a scale-free gene co-expression network was constructed to identify IPF-associated modules using the WGCNA package in R. The analysis followed a standardized pipeline: First, sample quality control was performed by hierarchical clustering to detect and remove outliers. Second, the optimal soft-thresholding power (β=5) was determined through dynamic tree-cutting to achieve a scale-free topology fit (scale-free $R^{\text{2}}>\text{0}.\text{85}$). Subsequently, gene modules were detected by performing hierarchical clustering on the topological overlap matrix (TOM), with parameters set to a minimum module size of 30 and a merge cut height of 0.25. Module-trait association analysis was then conducted by correlating module eigengenes with the IPF phenotype. Finally, hub genes within key modules were identified based on high intramodular connectivity, defined by a module membership measure (kME) greater than 0.8.

### Integration of 5-HMF targets and IPF-related genes

The intersection of the predicted targets of 5-HMF and the IPF-related genes determined by differential expression analysis and WGCNA was analyzed to identify the core candidate genes of the 5-HMF-IPF axis. These overlapping genes were considered candidate molecular links between 5-HMF and IPF, and visualized via Venn diagrams.

### Functional Enrichment Analysis

To elucidate potential mechanistic roles of 5-HMF in IPF development, functional enrichment analysis was performed. Gene Ontology (GO) terms, encompassing biological processes (BP), cellular components (CC), and molecular functions (MF), alongside KEGG pathways, were analyzed for the core candidate targets using the clusterProfiler package, with a significance threshold of P<0.05.

### Machine Learning-Based Prioritization of Candidate Genes

To prioritize candidate genes with discriminatory value for IPF, a comprehensive machine learning framework integrating multiple algorithms was established. Candidate genes derived from the intersection of predicted 5-HMF targets and IPF-associated genes were subjected to machine learning analysis using expression profile data from the IPF training cohort. Eleven classical machine learning algorithms (Lasso, SVM, RF, glmBoost, Stepglm, Ridge, Enet, GBM, LDA, XGBoost, and NaiveBayes) were employed to develop 127 predictive models. Hyperparameter optimization was conducted via 5-fold cross-validation, with stratified sampling for partitioning data into training and internal validation subsets. Model performance was rigorously assessed based on the area under the ROC curve (AUC), accuracy, and F1-score. Optimal single-model predictions were further integrated using a stacking ensemble learning strategy. High-confidence models (AUC > 0.9) were selected, and their feature genes were ranked by frequency to prioritize candidate genes. Finally, gene expression patterns were visualized using the pheatmap package.

### Model interpretation

To enhance the interpretability of the selected machine learning models, which inherently function as “black-box” predictors, we employed the SHapley Additive exPlanations (SHAP) framework. This algorithm quantifies the contribution of each input feature (gene) to individual predictions by calculating its SHAP value, thereby providing a transparent and consistent measure of feature importance. The application of SHAP enabled an interpretable assessment of the relative contribution of each candidate gene to the classification of IPF and control samples.

### Molecular docking analysis

To computationally explore potential interactions between 5-HMF and the prioritized target proteins, molecular blind docking simulations were performed using the CB-Dock2 web server. The three-dimensional structure of the 5-HMF ligand was downloaded from the PubChem database in SDF format and geometrically optimized via energy minimization using the MMFF94 force field. The crystal structures of human core target proteins were retrieved from the RCSB Protein Data Bank. All protein structures were preprocessed by removing water molecules, co-crystallized ligands, residual additives and counterions, while only single protein chains were retained for subsequent docking. Blind docking was carried out with default parameters, with docking boxes automatically centered on the top detected protein cavities. To verify the docking protocol, cognate ligands of each target were redocked into the corresponding protein structure, and the heavy-atom RMSD was calculated to evaluate docking reliability. Cross-docking of a classic inhibitor was additionally performed as a positive control for the ligand-free PLA2G1B protein. All docking poses and intermolecular interactions were finally analyzed and visualized using Discovery Studio 2019.

### Animal model of IPF

Adult male C57BL/6 J mice (20-25g) were sourced from the Animal Experimentation Department of Chongqing Medical University [License No. SCXK (Yu) 2022–0010]. All animal procedures were approved by the Ethics Committee of the Second Affiliated Hospital of Chongqing Medical University (Approval No. IACUC-SAHCQMU-2025-0021) and adhered to the ARRIVE guidelines.

After one week of acclimatization, all mice were randomized into control, bleomycin (BLM), and BLM + 5-HMF groups (n = 5 each). For intratracheal administration, mice were anesthetized with inhaled isoflurane (3% for induction and 1.5–2% for maintenance), and adequate depth of anesthesia was confirmed by the absence of the pedal withdrawal reflex. After adequate anesthesia was achieved, the trachea was visualized using a laryngoscope, and pulmonary fibrosis was induced by intratracheal instillation of BLM (5 mg/kg in 50 μL saline), whereas control mice received an equivalent volume of saline. No additional analgesics were administered. Following intratracheal instillation, mice were kept warm and closely monitored until they had fully recovered from anesthesia. Animals were subsequently monitored daily for general appearance, activity, body weight, respiratory status, and signs of pain or distress. Humane endpoints, including severe respiratory distress, inability to access food or water, markedly reduced mobility, or a body-weight loss exceeding 20% of the baseline value, were predefined to minimize unnecessary suffering. Animals reaching these criteria were to be euthanized immediately. 5-HMF (100 mg/kg), a dose selected with reference to previous oral pharmacokinetic and toxicological studies of 5-HMF in rodents [19,20], was administered by oral gavage once daily to mice in the BLM + 5-HMF group, whereas the control and BLM groups received an equivalent volume of vehicle. On day 21 after BLM administration, mice were deeply anesthetized with isoflurane (5%) and euthanized by cervical dislocation after loss of the pedal withdrawal reflex. Death was confirmed by the absence of respiration and heartbeat before lung tissues were harvested for subsequent analyses.

### Quantitative Real-Time PCR (qRT-PCR)

Total RNA was extracted from frozen lung tissues using TRIzol reagent following the manufacturer’s instructions. RNA concentration and purity were determined via spectrophotometry, and qualified RNA was reversely transcribed into complementary DNA (cDNA) using reverse transcription kits. Quantitative real‑time PCR was performed on a quantitative PCR instrument with SYBR Green PCR Master Mix. The transcription levels of *Hsp90aa1, Pla2g1b, Ca4, F10, Ca2, Esr2, Ptprb, Acta2 and Col1a1* were detected, with *Gapdh* selected as the internal reference gene. Relative mRNA expression was calculated by the 2^−ΔΔCt^ method, and the gene expression level of the control group was normalized to 1.

### Histopathological staining

Harvested lung tissues were fixed in 4% paraformaldehyde, embedded in paraffin and cut into 4 μm-thick serial sections for subsequent staining.

### Hematoxylin–eosin (HE) staining

After routine deparaffinization and gradient rehydration, tissue sections were stained with hematoxylin and eosin. Pulmonary pathological lesions including alveolar collapse, alveolar septum thickening and inflammatory cell infiltration were observed under light microscopy. The severity of lung injury was assessed using a semiquantitative histopathological scoring system. 6 non-overlapping fields were evaluated for each animal, and the mean score was used as the biological replicate for statistical analysis.

### Masson’s trichrome staining

Following deparaffinization and rehydration, sections were subjected to Masson trichrome staining according to kit protocols. Collagen fibers were stained blue, and collagen deposition was quantified using ImageJ software. The collagen-positive area was expressed as a percentage of the total tissue area. 6 non-overlapping fields were analyzed for each animal, and the mean value was used as the biological replicate for statistical analysis.

### Sirius Red staining

Deparaffinized sections were incubated with Sirius Red working solution to visualize collagen deposition. Sirius Red-positive areas were quantified using ImageJ software and expressed as a percentage of the total tissue area. 6 non-overlapping fields were analyzed for each animal, and the mean value was used as the biological replicate for statistical analysis.

All histological assessments and quantitative analyses were performed in a blinded manner.

### Statistical analysis

Statistical analyses were performed using GraphPad Prism 9.0. Data are presented as mean ± SD. For comparisons among three groups, data were assessed for normality and homogeneity of variance, followed by one-way analysis of variance (ANOVA). When the overall ANOVA was significant, Tukey’s multiple-comparisons test was performed for pairwise comparisons. Effect sizes for the overall ANOVA were estimated using eta squared (η²). The F statistics, corresponding P values, η² values, and Tukey-adjusted P values for the qRT-PCR and histological analyses are provided in S2 Table. A two-sided P < 0.05 was considered statistically significant.

## Results

### Identification of 5-HMF target proteins

The chemical structure of 5-HMF was obtained from the PubChem database (Fig 2A). Putative biological targets of 5-HMF were identified using three independent computational platforms: TargetNet (for bioactive molecule annotations), PharmMapper (based on reverse pharmacophore matching), and SwissTargetPrediction (using ligand similarity approaches). Following the consolidation of results and the elimination of duplicates, a total of 143 potential targets were retained for further analysis (Fig 2B).

**Fig 2:**
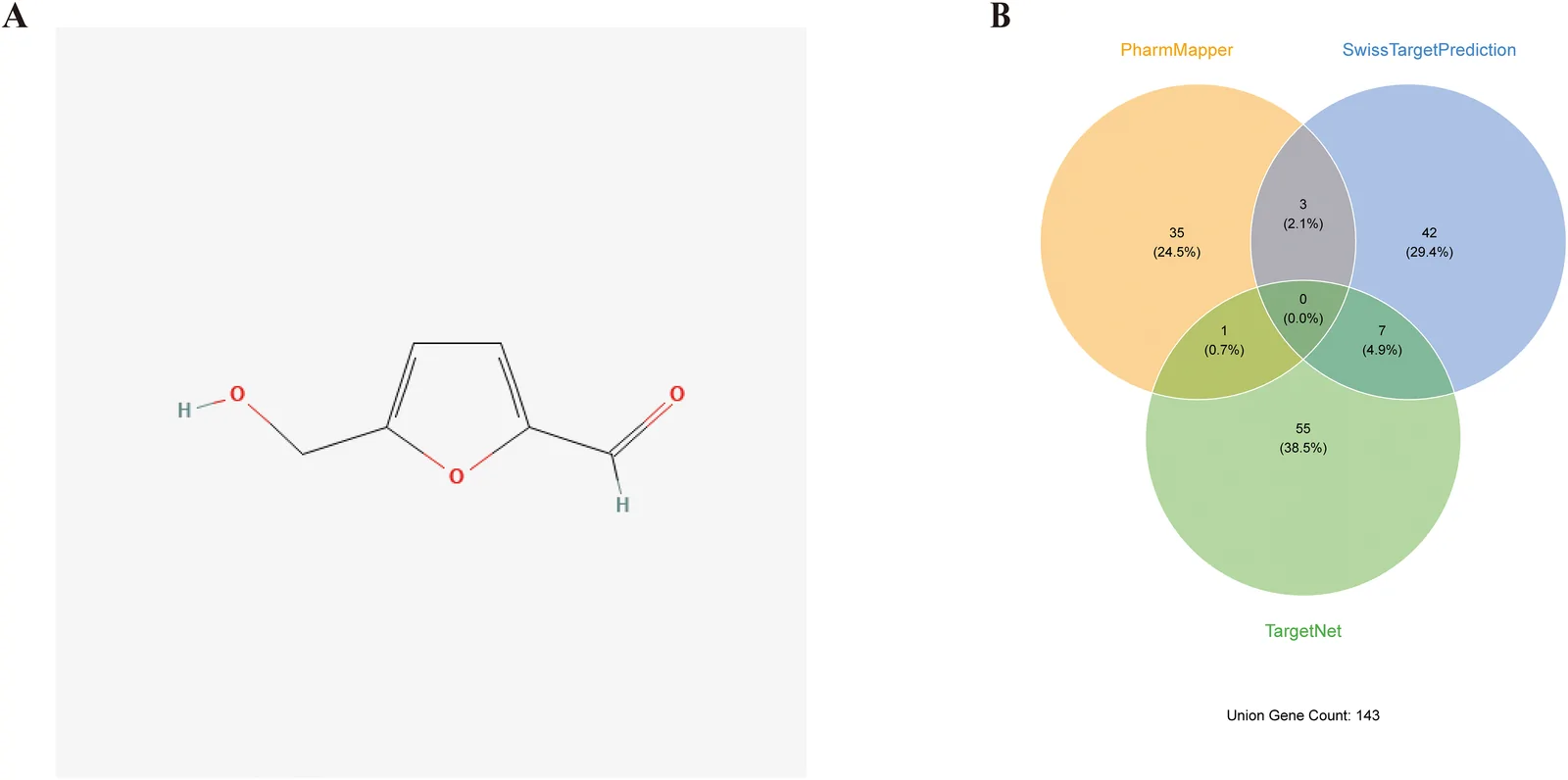
Identification of potential targets for 5-HMF. **(A)** The 2D chemical structure of 5-Hydroxymethylfurfural (5-HMF) retrieved from the PubChem database. **(B)** Venn diagram showing the intersection of 5-HMF targets predicted by three independent databases: PharmMapper, SwissTargetPrediction, and TargetNet. A total of 143 unique targets were identified.

### Identification of IPF-associated target genes

To minimize batch effects associated with the integration of multiple datasets, gene expression matrices from GSE53845, GSE110147, and GSE10667 were merged and subjected to stringent normalization procedures. PCA confirmed that normalization effectively improved data distribution, with the processed dataset exhibiting defined clustering patterns (Fig 3A, 3B). We observed that a total of 1346 differentially expressed genes (DEGs) were identified in IPF (P<0.05 and $|{\text{log}}_{\text{2}}FC|>\text{0}.\text{5}\text{8}\text{5}$), which were visualized using volcano plots and hierarchical clustering heatmaps (Fig 3C, 3D).

**Fig 3:**
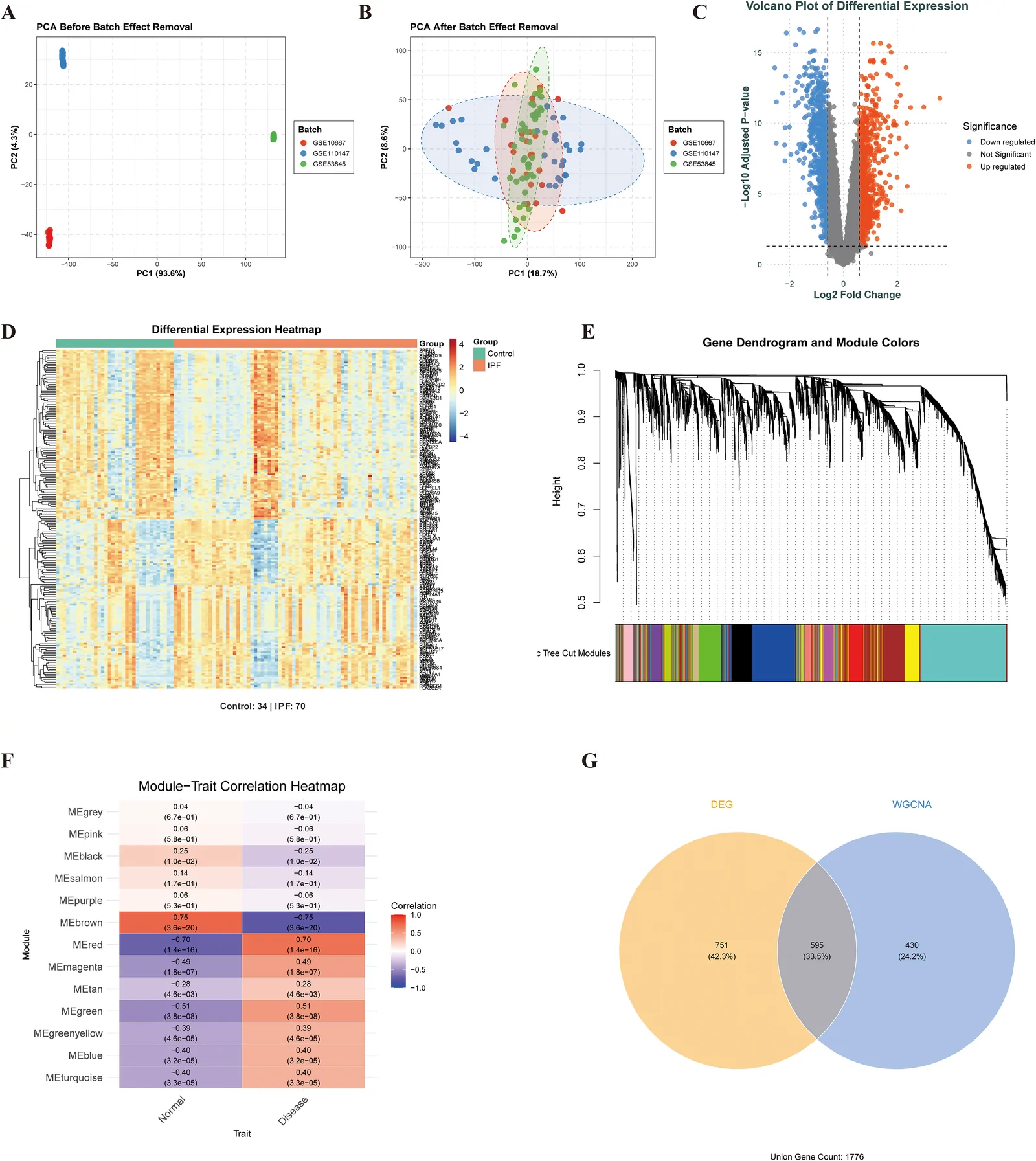
Identification of IPF-associated gene modules and differentially expressed genes (DEGs). **(A)** Principal Component Analysis (PCA) plot of gene expression profiles from three datasets before batch effect removal. **(B)** PCA plot after batch effect removal, demonstrating improved data integration. **(C)** Volcano plot visualizing DEGs between IPF and control samples. Red dots represent upregulated genes, and blue dots represent downregulated genes ($P<\text{0}.\text{05},|{\text{log}}_{\text{2}}FC|>\text{0}.\text{585}$). **(D)** Hierarchical clustering heatmap of DEGs showing distinct expression patterns between control and IPF groups. **(E)** Gene dendrogram and module assignment from Weighted Gene Co-expression Network Analysis (WGCNA). The color row underneath the dendrogram indicates the module membership of each gene. **(F)** Module-trait relationship heatmap. Each cell contains the correlation coefficient and P-value, highlighting modules significantly associated with the IPF disease state. **(G)** Venn diagram displaying the overlap between DEGs and WGCNA module genes, identifying 1776 robust IPF-associated genes.

For WGCNA, we first determined an appropriate soft-thresholding power (β) to construct a scale-free network. Evaluation of the scale-free topology fit index across power values confirmed β=5 as the optimal value that fulfilled the criterion (scale-free $R^{\text{2}}\geq \text{0}.\text{8}\text{5}$). Using this parameter, a topological overlap matrix (TOM) was generated, and hierarchical clustering was performed to delineate co-expression modules. This analytical workflow yielded discrete gene modules, each assigned a unique color for intuitive visualization (Fig 3E). Subsequent module-trait correlation analysis revealed that the Brown module exhibited the most significant correlation with the IPF phenotype (Fig 3F) and was therefore selected as the key disease-associated module.

Finally, by integrating the gene lists from the conventional differential expression analysis and the genes within the Brown module from WGCNA, we consolidated a robust set of 1776 high-confidence target genes related to IPF pathogenesis for further investigation (Fig 3G).

### Identification of 5-HMF-associated disease targets in IPF

Intersection analysis of the predicted 5-HMF protein targets and IPF-associated genes identified 12 overlapping targets, suggesting potential molecular links between 5-HMF and IPF (Fig 4A–4C). To functionally characterize these overlapping targets, Gene Ontology (GO) and Kyoto Encyclopedia of Genes and Genomes (KEGG) enrichment analyses were performed (Fig 4D, 4E). GO analysis highlighted enrichment in processes relevant to fibrotic progression. Key processes included the regulation of protein kinase B (Akt) signaling, which is a core mediator of the pro-fibrotic PI3K-Akt pathway that is clinically implicated in IPF pathogenesis and a promising therapeutic target [21,22].Additionally, enrichment in steroid hormone response was observed, implicating potential endocrine-immune crosstalk in disease pathogenesis. At the molecular function level, activities such as lipase activity and phospholipase activity were enriched, pointing toward altered lipid metabolism and membrane signaling in disease modulation. KEGG pathway analysis further supported involvement in specific disease-relevant mechanisms. Notably, the “Chemical carcinogenesis–receptor activation” pathway suggests a shared mode of action between chemical exposure and cellular injury responses. Additionally, enrichment in the “Estrogen signaling pathway” and “Folate biosynthesis” highlights potential roles of hormone-sensitive pathways and one-carbon metabolism in fibrotic tissue remodeling. Collectively, these results indicate that 5-HMF may influence IPF-relevant pathways through modulating specific signal transduction (such as PI3K-Akt, steroid hormone), cellular metabolism, and chemical stress response mechanisms.

**Fig 4:**
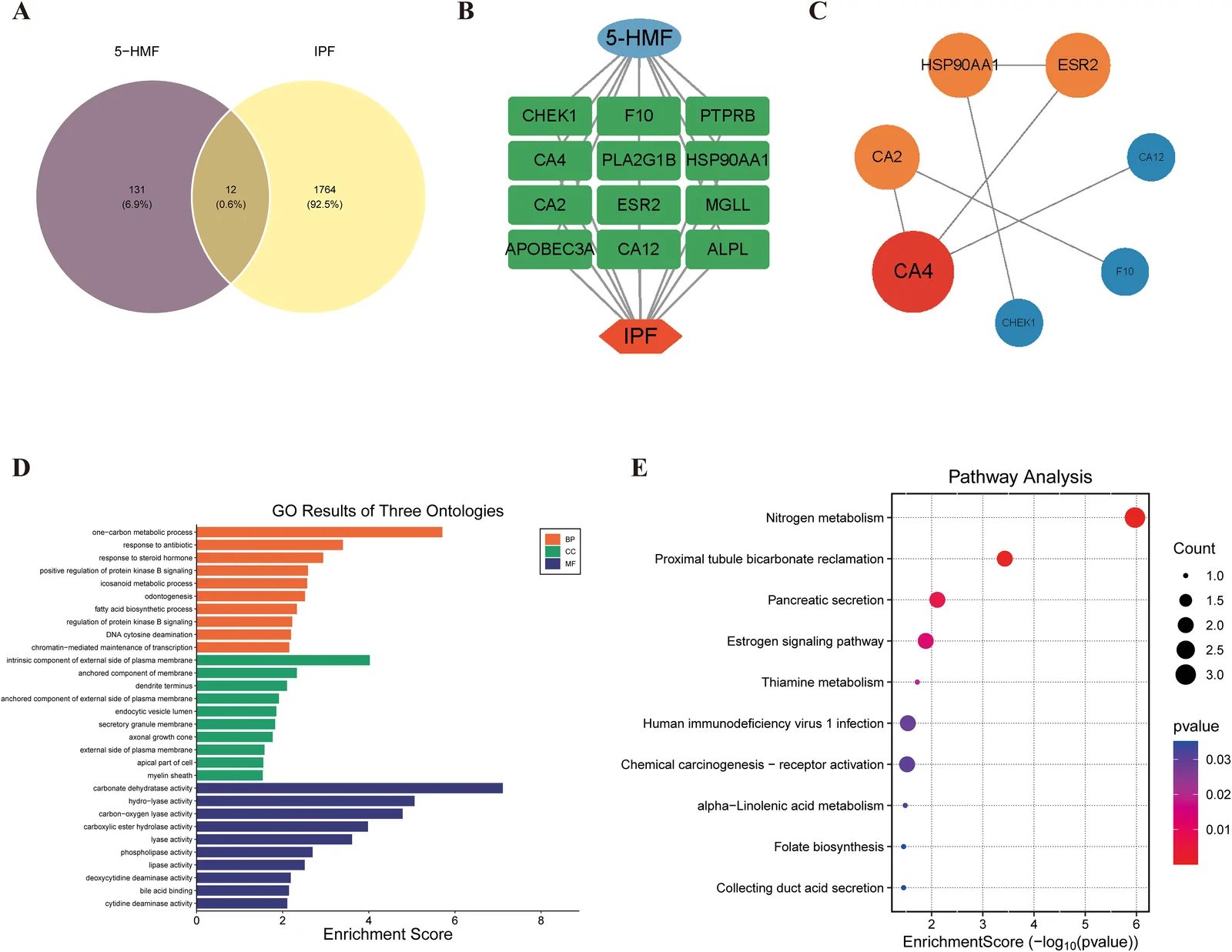
Characterization of the 5-HMF-IPF interaction network. **(A)** Venn diagram illustrating the intersection between 5-HMF-predicted targets (143) and IPF-associated genes (1776), resulting in 12 shared candidate targets. **(B)** The “5-HMF–Target–IPF” network diagram visualizing the interactions between 5-HMF and the 12 candidate genes implicated in IPF. **(C)** Protein-Protein Interaction (PPI) network of the 12 overlapping targets, constructed with a minimum interaction score of 0.4 for Homo sapiens. Node size corresponds to the degree of connectivity. **(D)** Gene Ontology (GO) enrichment analysis of the candidate targets, categorizing them into Biological Processes (BP), Cellular Components (CC), and Molecular Functions (MF). **(E)** Bubble plot of KEGG pathway enrichment analysis. The size of the bubble represents the gene count, and the color gradient indicates significance (P-value).

### Machine Learning-Based Prioritization and Interpretation of Candidate Genes for IPF Classification

To further prioritize candidate genes with discriminatory value for IPF, a comprehensive machine learning framework was established. By integrating 11 classical algorithms (Lasso, SVM, RF, glmBoost, Stepglm, Ridge, Enet, GBM, LDA, XGBoost, and NaiveBayes), we constructed and evaluated 127 classifier combinations. The RF + GBM (Random Forest integrated with Gradient Boosting Machine) ensemble model demonstrated superior predictive performance and stability, achieving high AUC values across the training cohort and two external validation datasets (GSE24206 and GSE70866) (Fig 5A). Based on feature importance ranking, seven candidate genes were prioritized: CA2, CA4, ESR2, F10, HSP90AA1, PLA2G1B, and PTPRB. The differential expression of these candidate genes in IPF was further confirmed (Fig 5D).

**Fig 5:**
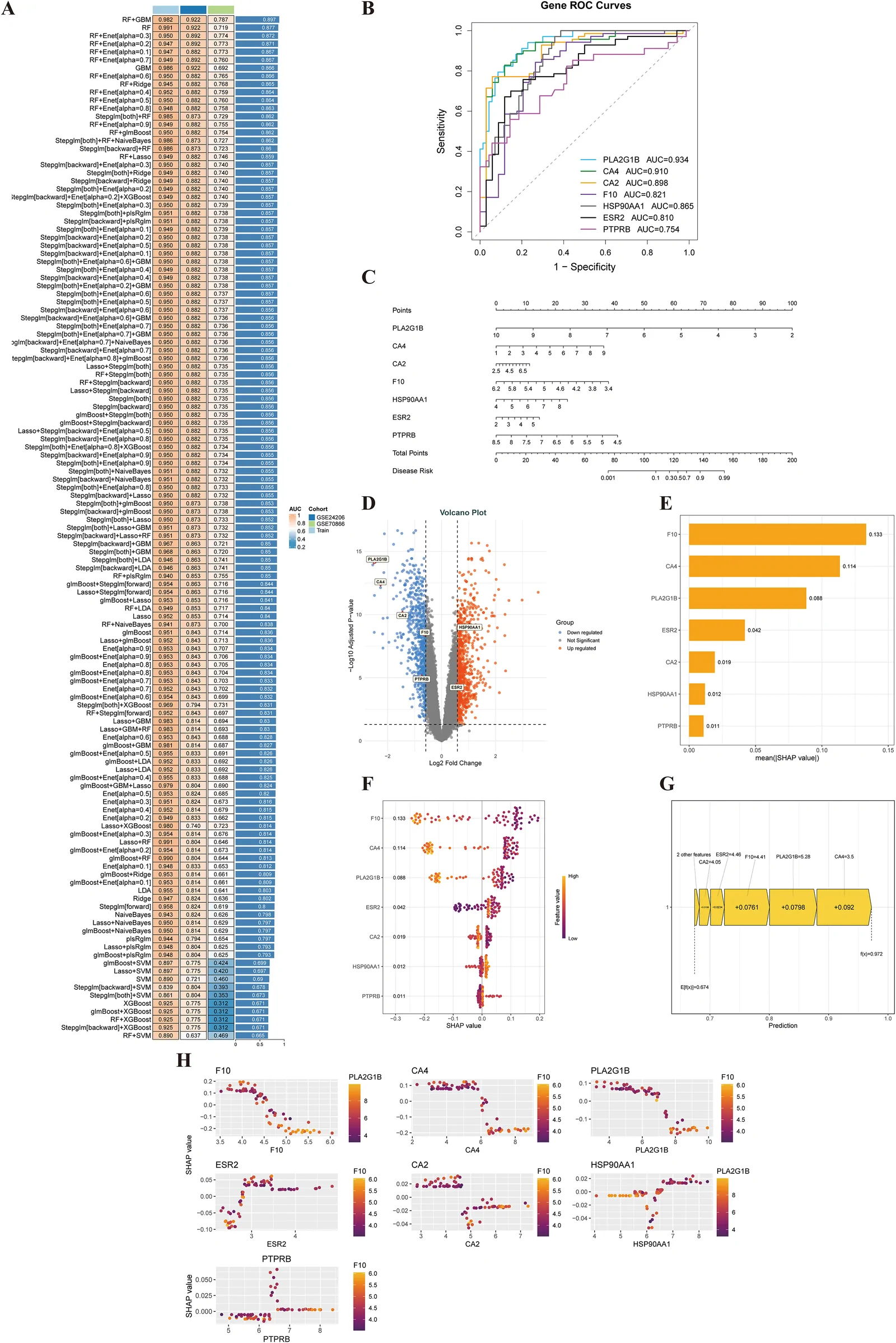
Machine learning-based prioritization and interpretation of candidate genes for IPF classification. **(A)** Performance comparison of 127 machine learning models across training and validation cohorts. The heatmap displays AUC values, with the RF + GBM ensemble model showing superior performance. **(B)** Receiver Operating Characteristic (ROC) curves for the seven core genes (PLA2G1B, CA4, CA2, F10, HSP90AA1, ESR2, PTPRB) in the training set. **(C)** Nomogram for predicting IPF classification based on the expression levels of the seven core genes. **(D)** Volcano plot highlighting the differential expression status of the seven core genes (labeled). **(E)** SHAP feature importance bar plot ranking the top genes (e.g., F10, CA4) based on their mean absolute SHAP values. **(F)** SHAP summary plot showing the distribution of SHAP values for each gene. Each dot represents a sample; color indicates feature value (red = high, purple = low). **(G)** SHAP force plot for an individual sample, illustrating how feature values push the prediction from the base value to the model output. **(H)** SHAP dependence plots for the core genes, revealing nonlinear relationships and interaction effects between features (e.g., F10 vs. PLA2G1B).

Individual ROC curve analysis revealed that these genes showed substantial discriminatory ability for IPF and control samples (Fig 5B), with PLA2G1B (AUC = 0.934) and CA4 (AUC = 0.910) exhibiting the highest AUC values, followed by CA2 (0.898), HSP90AA1 (0.865), F10 (0.821), ESR2 (0.810), and PTPRB (0.754). To further evaluate their combined discriminatory performance, a nomogram was constructed based on the expression levels of these seven candidate genes (Fig 5C).

Furthermore, the SHAP framework was employed to enhance model interpretability and quantify the relative contribution of each candidate gene to IPF classification. Global feature importance ranking identified F10 as the most influential feature in the classification model, followed by CA4 and PLA2G1B (Fig 5E). The SHAP summary plot visualized the directional impact of gene expression on model predictions; for instance, high expression of CA4 (indicated by red dots) was positively correlated with higher SHAP values, shifting the model prediction toward the IPF class, whereas the impact of F10 showed a more complex pattern (Fig 5F). Force-directed plots for individual samples demonstrated how the combination of these features contributed to individual model predictions (Fig 5G). Additionally, SHAP dependence plots revealed nonlinear relationships and potential interactions between genes, such as the expression-dependent effects of F10 and PLA2G1B, further characterizing the contributions and interactions of these candidate genes within the IPF classification model (Fig 5H).

### Molecular docking analysis of potential 5-HMF–target interactions

To explore potential molecular interactions between 5-HMF and the seven prioritized target proteins, molecular blind docking was performed against CA2, CA4, ESR2, F10, HSP90AA1, PLA2G1B, and PTPRB. 5-HMF yielded relatively weak Vina scores across the seven target proteins, ranging from −4.5 to −5.4 kcal/mol (Table 1). Docking simulations placed 5-HMF within the predicted orthosteric pockets of the seven target proteins, and visualization of the docking poses illustrated its predicted positioning and intermolecular contacts within these pockets (Fig 6). The docking protocol was further evaluated by cognate-ligand redocking, with all six ligand-bound receptors yielding heavy-atom RMSD values ≤2.0 Å (S1 Table and S1 Fig). Under the same docking conditions, the cognate ligands and the reference inhibitor MJ33 yielded more favorable Vina scores than 5-HMF, supporting the ability of the docking workflow to identify biologically relevant binding pockets. Collectively, these results support weak and relatively non-selective predicted interactions between 5-HMF and the prioritized target proteins and should be interpreted as structural hypotheses requiring further experimental validation.

**Table 1:** Blind-docking Vina scores of 5-HMF at the orthosteric pockets of seven core targets.

Ligand	Receptor	PDB ID	Resolution (Å)	Species	Pocket center (x, y, z)	Pocket size^a^	Vina score (kcal/mol)
**5-HMF**	**CA2**	**1CIL**	**1.6**	**Homo sapiens**	**−4, 3, 15**	**461**	**−5.0**
**5-HMF**	**CA4**	**5JN8**	**1.85**	**Homo sapiens**	**36, 9, 18**	**186**	**−5.0**
**5-HMF**	**ESR2**	**1QKM**	**1.8**	**Homo sapiens**	**28, 11, 110**	**950**	**−4.5**
**5-HMF**	**F10**	**1G2L**	**1.9**	**Homo sapiens**	**7, 11, 23**	**730**	**−5.2**
**5-HMF** **5-HMF** **5-HMF**	**HSP90AA1** **PLA2G1B** **PTPRB**	**1YET** **6Q42** **2H03**	**1.9** **1.9** **1.65**	**Homo sapiens** **Homo sapiens** **Homo sapiens**	**40, −48, 66** **7, 60, 137** **21, 4, 26**	**558** **401** **169**	**−4.6** **−5.4** **−5.0**

^a^CurPocket cavity volume reported as the number of cavity grid points (an integer), not in Å or Å³.

**Fig 6:**
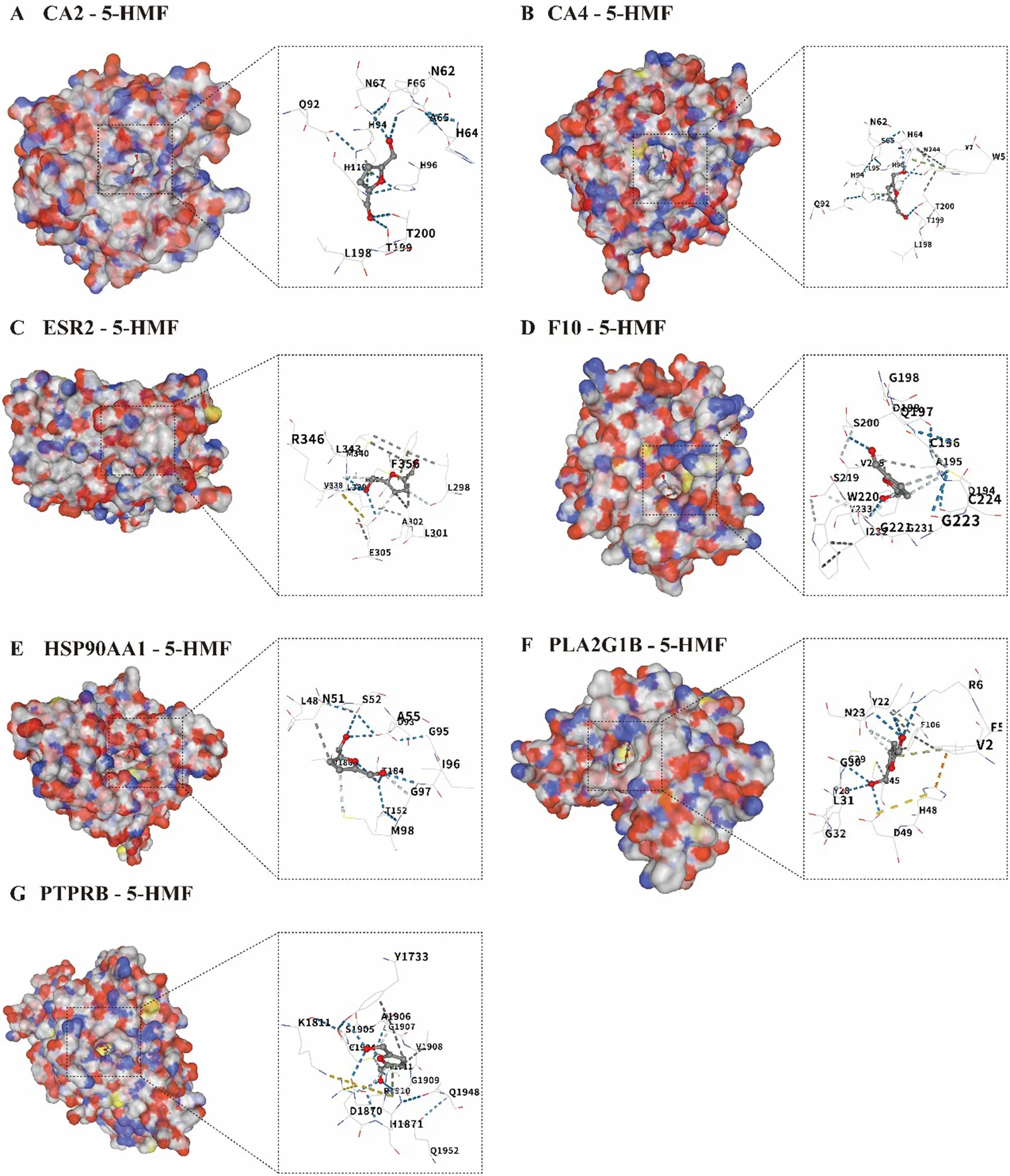
Predicted molecular docking poses of 5-HMF with the seven prioritized target proteins. **(A–G)** Visualization of the predicted docking poses and intermolecular contacts between 5-HMF and the seven core targets: (A) CA2, (B) CA4, (C) ESR2, (D) F10, (E) HSP90AA1, (F) PLA2G1B, and (G) PTPRB. The 3D surface models show the ligand occupying the binding pocket, while the 2D interaction diagrams detail specific hydrogen bonds and hydrophobic contacts with key amino acid residues.

### 5-HMF exacerbates BLM-induced pulmonary fibrosis

To investigate the profibrotic effects of 5-HMF in vivo, we established a bleomycin (BLM)-induced mouse pulmonary fibrosis model. Histopathological staining and qRT-PCR detection were performed to evaluate lung fibrotic lesions and the transcriptional expression of seven computationally prioritized candidate genes plus fibrotic markers Acta2 (α-SMA) and Col1a1.

qPCR data revealed that the candidate genes *Hsp90aa1, Pla2g1b, Ca4, F10*, as well as fibrotic markers *Acta2* and *Col1a1*, were significantly upregulated in the BLM group, and their expression was further increased upon 5-HMF treatment (Fig 7A). *Ca2* expression did not differ significantly between the control and BLM groups but was significantly increased in the BLM + 5-HMF group compared with the BLM group (Fig 7A). In contrast, no significant differences in *Esr2* or *Ptprb* expression were observed between the BLM and BLM + 5-HMF groups (Fig 7A). Collectively, five of the seven computationally prioritized candidate genes showed significant transcriptional changes following 5-HMF treatment in the BLM-induced pulmonary fibrosis model. Detailed statistical results, including F statistics, overall P values, effect sizes (η²), and Tukey-adjusted P values for all pairwise comparisons, are provided in S2 Table.

**Fig 7:**
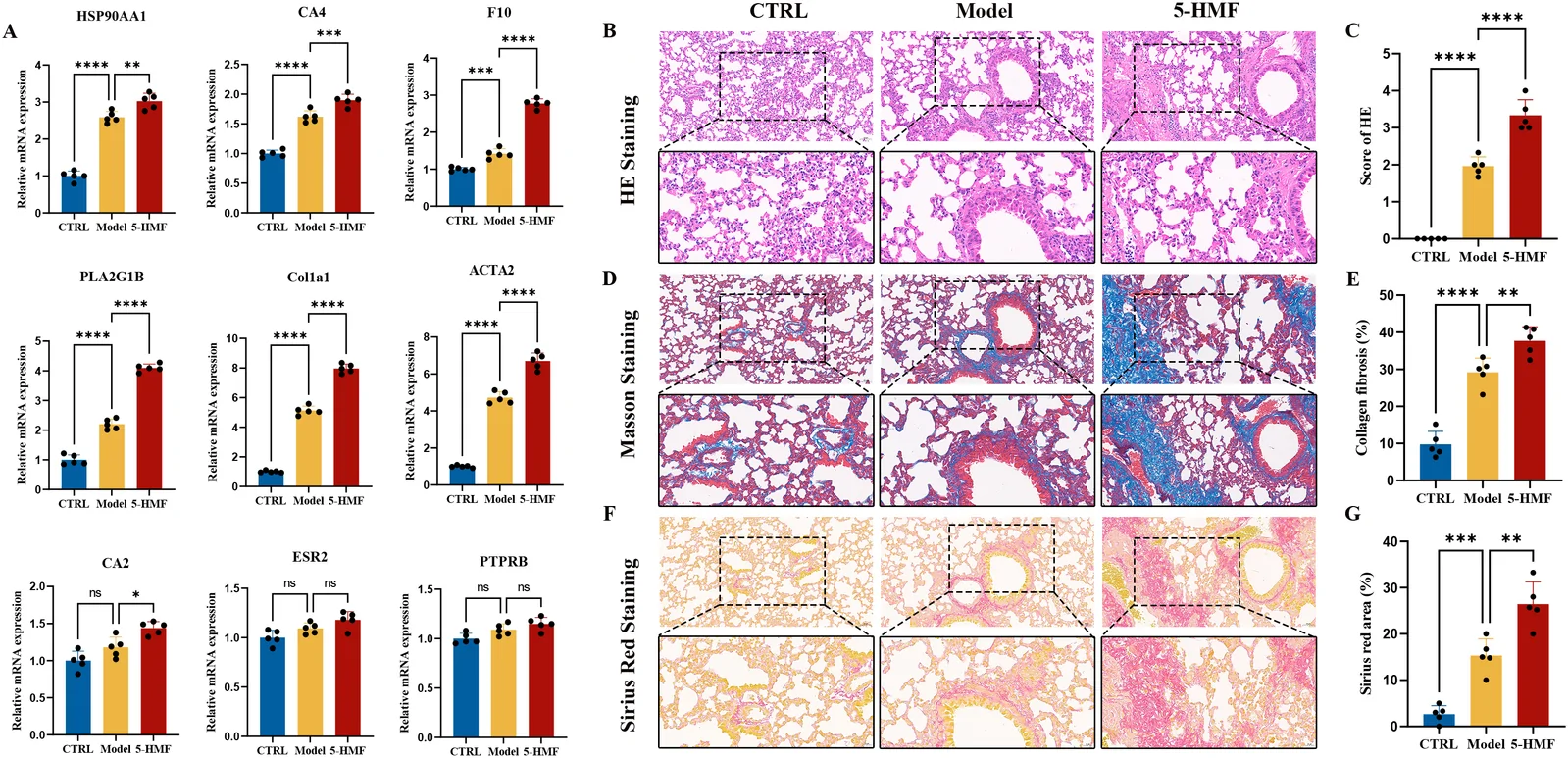
Effects of 5-HMF on BLM-induced pulmonary fibrosis in mice. **(A)** Relative mRNA expression of seven computationally prioritized candidate genes and fibrotic markers Acta2 (α-SMA), Col1a1 determined by qRT-PCR. **(B)** Representative H&E-stained lung sections and **(C)** quantitative histopathological scores. **(D)** Representative Masson’s trichrome-stained lung sections and **(E)** quantitative analysis of collagen-positive areas. **(F)** Representative Sirius Red-stained lung sections and **(G)** quantitative analysis of Sirius Red-positive areas. Data are presented as mean ± SD (n = 5 per group). Statistical comparisons were performed using one-way ANOVA followed by Tukey’s multiple-comparisons test. ns: no significance, *P < 0.05, **P < 0.01, ***P < 0.001, ****P < 0.0001.

Histological examination further demonstrated that 5-HMF aggravated BLM-induced lung injury and fibrosis (Fig 7B–7G). Quantitative analysis of H&E-stained sections showed a significantly higher histopathological score in the BLM + 5-HMF group than in the BLM group (Tukey-adjusted P < 0.0001; Fig 7B, 7C). Consistently, quantitative analyses of Masson’s trichrome and Sirius Red staining demonstrated significantly greater collagen deposition in the BLM + 5-HMF group than in the BLM group (adjusted P = 0.0088 and P = 0.0011, respectively; Fig 7D–7G). Detailed F statistics, effect sizes, and adjusted P values are provided in S2 Table.

Collectively, these in vivo findings demonstrate that 5-HMF exacerbates BLM-induced pulmonary fibrosis and is accompanied by transcriptional changes in several computationally prioritized candidate genes.

## Discussion

This study investigated the potential molecular links between 5-HMF and pulmonary fibrosis using an integrative framework combining network toxicology, ensemble machine learning, molecular docking, and in vivo validation. We prioritized a seven-gene signature (CA2, CA4, ESR2, F10, HSP90AA1, PLA2G1B, and PTPRB) associated with IPF-related molecular alterations and identified several potentially relevant pathways. Importantly, in vivo experiments demonstrated that 5-HMF aggravated histopathological injury, collagen deposition, and fibrosis-related gene expression in mice with BLM-induced pulmonary fibrosis. These findings support a potential role of 5-HMF in exacerbating fibrotic lung injury.

IPF is characterized by repetitive alveolar epithelial cell injury, aberrant repair, and excessive extracellular matrix deposition [23–25]. Our functional enrichment analysis indicated that 5-HMF targets were significantly enriched in “Chemical carcinogenesis–receptor activation” and oxidative stress-related pathways. This aligns closely with the “multiple-hit” hypothesis of IPF, where environmental insults drive epithelial senescence and the formation of a pro-fibrotic microenvironment [26]. Notably, the identification of F10 (Coagulation Factor X) as the top contributor in our SHAP model highlights the critical role of the coagulation cascade in fibrosis. Activated Factor X is known to stimulate fibroblast proliferation and collagen production via Protease-Activated Receptors (PARs), linking vascular injury and dietary pro-coagulant states to lung remodeling. Furthermore, PLA2G1B (Phospholipase A2 Group IB) suggests a mechanism involving lipid metabolism dysregulation and arachidonic acid-mediated inflammation, which are potent drivers of fibrotic progression.

The identification of HSP90AA1 (Heat Shock Protein 90 Alpha Family Class A Member 1) underscores the importance of proteostasis in fibrosis. HSP90 serves as a chaperone for TGF-β signaling components; its inhibition has been shown to attenuate myofibroblast transformation, making it a viable therapeutic target [27–29]. ESR2 (Estrogen Receptor 2) was also identified as a candidate gene in our computational analysis, suggesting a potential link between estrogen signaling and IPF. However, Esr2 expression was not significantly altered by 5-HMF treatment in our in vivo experiments, and only male mice were included in the present study. Therefore, the potential involvement of ESR2 and estrogen signaling remains hypothetical and requires validation in both sexes in future studies.

Additionally, members of the carbonic anhydrase family (CA2 and CA4) were prioritized as candidate genes with discriminatory value for IPF. Carbonic anhydrases regulate pH homeostasis and CO2 hydration, influencing cell metabolism and extracellular matrix acidification [30]. Recent studies indicate that CA2 is upregulated in lung fibroblasts and promotes fibrosis by modulating the TGF-β1 signaling pathway [31], while CA4, primarily localized in the pulmonary capillary endothelium, may participate in hypoxia-induced vascular remodeling [32]. Blind docking placed 5-HMF in the orthosteric pockets of CA2 and CA4 with weak and non-selective Vina scores (−5.0 kcal/mol for both); whether 5-HMF can perturb pulmonary pH regulation therefore remains speculative and requires enzyme-inhibition assays.

From a methodological perspective, this study integrates network toxicology, machine learning, molecular docking, and in vivo experiments within a complementary analytical framework. The ensemble machine learning strategy combined with SHAP interpretation enabled the prioritization and interpretation of candidate genes with discriminatory value for IPF, while molecular docking generated structural hypotheses regarding their potential interactions with 5-HMF. The subsequent in vivo experiments further evaluated the fibrotic phenotype and transcriptional changes in the prioritized candidate genes.

The exposure level used in the present study should also be considered when interpreting the translational relevance of our findings. The 5-HMF dose of 100 mg/kg/day was selected with reference to previous oral pharmacokinetic and toxicological studies in rodents [19,20]. Based on body-surface-area scaling, this dose corresponds to an approximate human equivalent dose of 8.1 mg/kg/day, or approximately 486 mg/day for a 60-kg adult [33]. In comparison, estimated human dietary intake of 5-HMF is generally approximately 4–30 mg/person/day, although substantially higher intake of up to approximately 350 mg/day has been reported under specific dietary conditions [20]. 5-HMF is widely present in heat-processed foods and beverages, including coffee, honey, and fruit juices, although its concentration varies considerably according to food type, processing, and storage conditions. Thus, the human equivalent of the exposure used in our mouse model is approximately one to two orders of magnitude higher than typical dietary intake and should be regarded as an experimental exposure rather than a direct simulation of habitual dietary exposure. Accordingly, the present findings demonstrate that 5-HMF exacerbates BLM-induced fibrotic lung injury under the tested exposure conditions, whereas extrapolation of these findings to human dietary risk requires further investigation.

However, several limitations must be acknowledged. First, the in vivo experiment did not include a 5-HMF-only group. Therefore, the present findings demonstrate that 5-HMF exacerbates BLM-induced pulmonary fibrosis but do not establish whether 5-HMF alone is sufficient to initiate fibrotic lung pathology. Future studies incorporating a 5-HMF-only group and different exposure durations will be necessary to address its potential independent pulmonary effects. Second, although in vivo validation was performed, the mechanistic relationship between 5-HMF and the identified targets remains largely inferential and requires further molecular investigation. In particular, molecular docking provides computational support for potential binding hypotheses rather than definitive evidence of direct molecular interactions, and docking scores cannot accurately determine binding affinity. Further biochemical and biophysical studies are therefore required to validate these predicted interactions. Third, circulating and lung concentrations of 5-HMF were not measured, leaving the actual systemic and pulmonary exposure under our dosing regimen unclear. Future pharmacokinetic studies measuring 5-HMF levels in plasma and lung tissue are needed to establish the exposure–effect relationship and assess the translational relevance of our findings. Finally, the cell-type-specific expression and functional roles of the identified targets require further validation using approaches such as single-cell or spatial transcriptomic analyses. In addition, only male mice were used in the present study, which limits the interpretation of potential sex-dependent mechanisms, particularly those involving ESR2 and estrogen signaling. Future studies including both male and female animals are required to investigate potential sex-specific effects of 5-HMF.

## Conclusion

In conclusion, this integrative study identifies potential molecular links between 5-HMF and pulmonary fibrosis and provides in vivo evidence that 5-HMF can exacerbate BLM-induced fibrotic lung injury. Our findings further identify F10, HSP90AA1, and CA2/CA4 as candidate molecular targets potentially associated with the effects of 5-HMF in pulmonary fibrosis. These identified targets furnish a prioritized framework for subsequent experimental toxicology and may inform future considerations in dietary exposure assessment for populations susceptible to interstitial lung diseases.

## Supporting information

S1 Table Cognate-ligand redocking validation and positive-control docking performed on CB-Dock2. (DOCX)

S2 Table Detailed statistical analysis of qRT-PCR and histological data in the in vivo experiment. (DOCX)

S1 Fig Docking validation using cognate-ligand redocking and positive-control docking. (DOCX)
