# Supplementary material for: Integrative network toxicology and machine learning reveal the potential role of 5-hydroxymethylfurfural in exacerbating pulmonary fibrosis
Source: PLoS One. 2026 Nov 2;21(11):e0361700. doi: 10.1371/journal.pone.0361700 (PMC13727047; doi:10.1371/journal.pone.0361700)
Supplement: Supplementary file 1 [file pone.0361700.s001.docx]

**Table S1. Cognate-ligand redocking validation and positive-control docking performed on CB-Dock2**

| **Ligand** | **Receptor** | **PDB ID** | **Type** | **Pocket center (x, y, z)** | **Pocket size** | **Vina score (kcal/mol)** | **Heavy-atom RMSD (Å)** | **Pass ≤ 2.0 Å** |
| --- | --- | --- | --- | --- | --- | --- | --- | --- |
| **ETS** | **CA2** | **1CIL** | **cognate redock** | **-4, 3, 15** | **461** | **-7.2** | **0.44** | **Yes** |
| **AZM** | **CA4** | **5JN8** | **cognate redock** | **36, 9, 18** | **186** | **-6.4** | **1.05** | **Yes** |
| **GEN** | **ESR2** | **1QKM** | **cognate redock** | **28, 11, 110** | **950** | **-9.3** | **0.30** | **Yes** |
| **T87** | **F10** | **1G2L** | **cognate redock** | **7, 11, 23** | **730** | **-9.7** | **1.17** | **Yes** |
| **GDM**  **MJ33 (MJI)**  **3UN** | **HSP90AA1**  **PLA2G1B**  **PTPRB** | **1YET**  **6Q42**  **2H03** | **cognate redock positive control^a^**  **cognate redock** | **40, -48, 66**  **7, 60, 137**  **21, 4, 26** | **558**  **401**  **169** | **-7.2**  **-6.1**  **-7.3** | **0.33**  **N/A**  **1.43** | **Yes**  **N/A**  **Yes** |

**Table notes:**

- a. 6Q42 contains no co-crystallized ligand. MJ33 co-crystallized with porcine group-IB PLA2 (PDB 1FX9), was cross-docked into human 6Q42 as a positive control and localized to the His48–Asp49–Gly30 catalytic pocket; no RMSD is applicable.
- Full names of the ligands: ETS, (4S-trans)-4-(ethylamino)-5,6-dihydro-6-methyl-4H-thieno[2,3-b]thiopyran-2-sulfonamide 7,7-dioxide; AZM, acetazolamide (5-acetamido-1,3,4-thiadiazole-2-sulfonamide); GEN, genistein; T87, a synthetic benzimidazole-based factor Xa inhibitor; GDM, geldanamycin; 3UN, (4-{4-[(tert-butoxycarbonyl)amino]-2,2-bis(ethoxycarbonyl)butyl}phenyl)sulfamic acid.
