# Supplementary material for: Integrative network toxicology and machine learning reveal the potential role of 5-hydroxymethylfurfural in exacerbating pulmonary fibrosis
Source: PLoS One. 2026 Nov 2;21(11):e0361700. doi: 10.1371/journal.pone.0361700 (PMC13727047; doi:10.1371/journal.pone.0361700)
Supplement: Supplementary file 2 [file pone.0361700.s002.docx]

**Table S2. Detailed statistical analysis of qRT-PCR and histological data in the in vivo experiment**

| **Outcome** | **F (2, 12)** | **Overall P value** | **η²** | **CTRL vs BLM, adjusted P** | **CTRL vs BLM+5-HMF, adjusted P** | **BLM vs BLM+5-HMF, adjusted P** |
| --- | --- | --- | --- | --- | --- | --- |
| **qRT-PCR analysis** | | | | | | |
| *Hsp90aa1* | 196.783 | <0.0001 | 0.970 | <0.0001 | <0.0001 | 0.0042 |
| *Pla2g1b* | 483.191 | <0.0001 | 0.988 | <0.0001 | <0.0001 | <0.0001 |
| *Ca4* | 134.502 | <0.0001 | 0.957 | <0.0001 | <0.0001 | 0.0009 |
| *F10* | 360.312 | <0.0001 | 0.984 | 0.0002 | <0.0001 | <0.0001 |
| *Ca2* | 17.250 | 0.0003 | 0.742 | 0.0810 | 0.0002 | 0.0123 |
| *Esr2* | 7.632 | 0.0073 | 0.560 | 0.1451 | 0.0055 | 0.1908 |
| *Ptprb* | 7.844 | 0.0066 | 0.567 | 0.0851 | 0.0052 | 0.2937 |
| *Acta2* | 419.299 | <0.0001 | 0.986 | <0.0001 | <0.0001 | <0.0001 |
| *Col1a1* | 981.267 | <0.0001 | 0.994 | <0.0001 | <0.0001 | <0.0001 |
| **Histological analysis** | | | | | | |
| H&E histopathological score | 174.285 | <0.0001 | 0.967 | <0.0001 | <0.0001 | <0.0001 |
| Masson's trichrome staining | 75.791 | <0.0001 | 0.927 | <0.0001 | <0.0001 | 0.0088 |
| Sirius Red staining | 53.404 | <0.0001 | 0.899 | 0.0004 | <0.0001 | 0.0011 |

Note: Data were analyzed using one-way ANOVA followed by Tukey’s multiple-comparisons test (n = 5 per group). η² denotes eta squared and represents the overall ANOVA effect size. Pairwise P values are Tukey-adjusted P values. A two-sided P < 0.05 was considered statistically significant.
