# Supplementary material for: Integrative network toxicology and machine learning reveal the potential role of 5-hydroxymethylfurfural in exacerbating pulmonary fibrosis
Source: PLoS One. 2026 Nov 2;21(11):e0361700. doi: 10.1371/journal.pone.0361700 (PMC13727047; doi:10.1371/journal.pone.0361700)
Supplement: Supplementary file 3 [file pone.0361700.s003.docx]

**Figure S1. Docking validation using cognate-ligand redocking and positive-control docking.**
Representative docking poses used to evaluate the docking protocol for the seven prioritized target proteins. Cognate ligands were redocked into their corresponding protein structures for (A) CA2–ETS, (B) CA4–AZM, (C) ESR2–GEN, (D) F10–T87, (E) HSP90AA1–GDM, and (G) PTPRB–3UN. Because the human PLA2G1B structure (PDB ID: 6Q42) lacks a co-crystallized ligand, the reference inhibitor MJ33 was cross-docked as a positive control (F). The left panels show the predicted ligand-binding poses within the protein pockets, and the enlarged right panels illustrate the corresponding protein–ligand interactions. For the six cognate-ligand redocking analyses, all heavy-atom RMSD values between the redocked and crystallographic poses were ≤2.0 Å. Detailed Vina scores and RMSD values are provided in Table S1.
